# Sorghum Growth Promotion by *Paraburkholderia tropica* and *Herbaspirillum frisingense*: Putative Mechanisms Revealed by Genomics and Metagenomics

**DOI:** 10.3390/microorganisms8050725

**Published:** 2020-05-13

**Authors:** Eiko E. Kuramae, Stan Derksen, Thiago R. Schlemper, Maurício R. Dimitrov, Ohana Y. A. Costa, Adriana P. D. da Silveira

**Affiliations:** 1 Netherlands Institute of Ecology (NIOO-KNAW), Microbial Ecology Department, Droevendaalsesteeg 10, 6708 PB Wageningen, The Netherlands; stan.derksen.1997@outlook.com (S.D.); trschlemper@gmail.com (T.R.S.); m.dimitrov@nioo.knaw.nl (M.R.D.); o.costa@nioo.knaw.nl (O.Y.A.C.); 2 Utrecht University, Institute of Environmental Biology, Ecology and biodiversity, 3508 TC Utrecht, The Netherlands; 3 Center of Soil and Environmental Resources, Agronomic Institute of Campinas (IAC), Av. Barão de Itapura 1481, 13020-902 Campinas, Brazil

**Keywords:** plant growth-promoting rhizobacteria, signal processing, biofilm, nutrient acquisition, comparative genomics, *Paraburkholderia tropica*, *Herbaspirillum frisingense*

## Abstract

Bacteria from the genera *Paraburkholderia* and *Herbaspirillum* can promote the growth of *Sorghum bicolor*, but the underlying mechanisms are not yet known. In a pot experiment, sorghum plants grown on sterilized substrate were inoculated with *Paraburkholderia tropica* strain IAC/BECa 135 and *Herbaspirillum* *frisingense* strain IAC/BECa 152 under phosphate-deficient conditions. These strains significantly increased *Sorghum bicolor* cultivar SRN-39 root and shoot biomass. Shotgun metagenomic analysis of the rhizosphere revealed successful colonization by both strains; however, the incidence of colonization was higher in plants inoculated with *P. tropica* strain IAC/BECa 135 than in those inoculated with *H. frisingense* strain IAC/BECa 152. Conversely, plants inoculated with *H. frisingense* strain IAC/BECa 152 showed the highest increase in biomass. Genomic analysis of the two inoculants implied a high degree of rhizosphere fitness of *P. tropica* strain IAC/BECa 135 through environmental signal processing, biofilm formation, and nutrient acquisition. Both genomes contained genes related to plant growth-promoting bacterial (PGPB) traits, including genes related to indole-3-acetate (IAA) synthesis, nitrogen fixation, nodulation, siderophore production, and phosphate solubilization, although the *P. tropica* strain IAC/BECa 135 genome contained a slightly more extensive repertoire. This study provides evidence that complementary mechanisms of growth promotion in Sorghum might occur, i.e., that *P. tropica* strain IAC/BECa 135 acts in the rhizosphere and increases the availability of nutrients, while *H. frisingense* strain IAC/BECa 152 influences plant hormone signaling. While the functional and taxonomic profiles of the rhizobiomes were similar in all treatments, significant differences in plant biomass were observed, indicating that the rhizobiome and the endophytic microbial community may play equally important roles in the complicated plant-microbial interplay underlying increased host plant growth.

## 1. Introduction

An essential step in plant–microbe interactions is the colonization of the rhizosphere by microorganisms. The rhizobiome is shaped by soil characteristics such as moisture, nutrient availability, texture, pH, and climate, but to an even greater extent by the host plant [1,2,3,4,5]. The host plant shapes the rhizobiome by secreting amino acids, sugars, carbohydrates, vitamins, and enzymes, as well as some plant hormones and flavonoids, which may act as signaling molecules in intra-kingdom communication [6,7]. Remarkably, between 5% and 21% of all photosynthetically fixed carbon is transferred to the rhizosphere through root exudates. These secretions alter the chemical and physical properties of the soil and thus regulate the microbial community occupying the rhizosphere [8]. The microbial community occupying the rhizosphere (the rhizobiome) can have a negative or positive effect on plant growth, depending on its composition.

The first step in colonization is the migration of bacteria to the rhizosphere. To attract the desired microbes, the plant must exude substances that act as chemoattractants. The chemoattractant role of exudates was thought to be plant-specific [9], but recent work demonstrated that it is even cultivar-specific [5]. Following successful migration to the rhizosphere, bacteria must attach to the roots. This process can be separated into two phases, namely, the “adsorption” phase and the “anchoring” phase. During the adsorption phase, a weak interaction is established based on the biochemical properties of both the roots and bacteria, such as electrostatic charge, polarity, and hydrophobicity [10]. After this initial attachment, the bacteria secrete extracellular polymeric substances (EPS) consisting of polysaccharides and adhesive proteins to create a matrix in which they can embed themselves through external appendages [11]. The formation of this biofilm is often (partially) regulated by a system called quorum sensing (QS). QS allows bacteria to communicate and regulate gene expression based on cell density. There are many different types of QS systems, but in general, small signal molecules, termed autoinducers because they are usually constitutively expressed, are secreted by the bacteria and accumulate in the extracellular environment as the population size increases. Upon reaching a threshold concentration, the bacteria sense the surrounding cell population, and regulation of specific genes is initiated [12]. The bacteria can release antibacterial and antifungal compounds into the biofilm matrix to create an optimal environment. In nutrient-deficient conditions, the associated bacteria can also release nutrients that can be absorbed by the host plant, such as fixed nitrogen or solubilized phosphate [13].

Plant growth-promoting rhizobacteria (PGPR) are naturally occurring soil-borne bacteria that associate with plant roots and promote plant growth via multiple mechanisms. (i) Nitrogen (N) is the most vital nutrient for plant development. Although N is abundant in the atmosphere (roughly 78% N_2_), this form of nitrogen is inaccessible to plants. N fixation by nitrogen-fixing microorganisms converts atmospheric N to ammonia, which is accessible to plants, via nitrogenase [14,15]. (ii) The second most vital nutrient after N is phosphate (P). Although abundant in soils [16], P is not available for plants, particularly in tropical soils. Plants can only uptake two soluble forms of P, namely, monobasic (H_2_PO_4_) and dibasic (HPO_4_^2−^) ions [17]. Upon exposure to soil, phosphate is insolubilized via various reactions whose rates depend on several chemical properties of the soil [18]. P solubilization by phosphate-solubilizing microorganisms (PSM) produces low-molecular-weight organic acids that solubilize mineralized phosphate and thus increase the amount of phosphate available to plants [16]. (iii) Plants need iron (Fe) to produce chlorophyll, a pigment required for photosynthesis. In aerobic environments, such as most farmland soils, Fe is present mostly as Fe^3+^, which tends to form insoluble hydroxides, making it inaccessible to plants [19]. Bacteria acquire iron by secreting soluble iron chelators termed siderophores, which have high association constants for complexing iron [16]. Siderophore-producing bacteria thus increase the amount of soluble iron that can be readily taken up by plants. In addition to the direct effect of increasing the iron concentration around the roots, siderophore-producing bacteria can inhibit pathogen growth [20]. (iv) Auxin is an important plant hormone implicated in the wound response, root growth and development, fruit growth, flowering, and many other important processes [21,22,23,24]. Many microorganisms found in the rhizosphere are able to produce auxin as a secondary metabolite [25]. (v) Ethylene is a plant growth regulator as well as a stress hormone. The endogenous level of ethylene is significantly increased under stress conditions such as salinity, drought, water logging, heavy metals, and pathogens, which negatively impacts plant growth and crop yield [26]. Some PGPR possess the enzyme 1-aminocyclopropane-1-carboxylate (ACC) deaminase, which converts the ethylene precursor ACC to 2-oxobutanoate and NH_3_ [27]. PGPR absorb ACC from the host plant and break it down, thereby reducing the effect of stress on plant growth and increasing resistance to stress-inducing conditions [28,29].

Several species of PGPR were shown to enhance the growth of sorghum, a multifunctional crop grown for food, feed, fiber, and fuel [30]. In addition to growth promotion, PGPR can offer tolerance to pathogenic fungi, bacteria, or parasitic plants, and are able to protect the plant from abiotic stresses such as drought [30,31,32]. Due to their positive effects on plant growth and their natural occurrence, PGPR can be used as a bio-fertilizer to partially or completely replace chemical fertilizers and reduce the associated environmental hazards. Bacteria of the genera *Herbaspirillum* and *Paraburkholderia* (previously bacteria of the genus *Burkholderia* that were split into the environmental and pathogenic genera *Paraburkholderia* and *Burkholderia*, respectively [33]) were identified as PGPR in several C4 plants, such as sugarcane, maize, rice, and numerous grasses [34,35,36,37]. A study assessing the sorghum plant growth-promoting abilities of several bacteria isolated from sugarcane roots identified *Herbaspirillum frisingense* strain IAC/BECa 152 and *Paraburkholderia tropica* strain IAC/BECa 135 as high promoters [38,39]. Increased shoot and root biomass were observed, but the effects of the inoculum treatment on the remaining rhizosphere microbial community were not elucidated. To date, PGPR research mostly focused on single microbes and the effects of their presence on the host phenotype [38,40,41]. Recently, a number of studies highlighted the importance of microbe–microbe and microbe–host interactions for successful colonization of the rhizosphere and subsequent growth promotion [7,38,42,43]. In this study, we compare the effects of inoculation with two PGPR, *P. tropica* strain IAC/BECa 135 and *H. frisingense* strain IAC/BECa 152, on the rhizobiome of *Sorghum bicolor* cultivar SRN-39 under P starvation. First, we completely sequenced both bacterial genomes; second, we identified genomic traits of the inoculants explaining their different effects on plants and rhizobiomes; finally, we analyzed the PGPR capabilities of the communities as a whole.

## 2. Materials and Methods

### 2.1. Bacterial Genome Sequencing and Assembly

*P. tropica* strain IAC/BECa 135 and *H. frisingense* strain IAC/BECa 152 were previously isolated from sugarcane roots. Each bacterial strain inoculum was cultivated in dextrose, yeast, and glutamate (DYGS) liquid medium at 30 °C. The growth curve was determined, and the cells were harvested at exponential growth. The medium containing the cells was centrifuged (10,000 × g), and genomic DNA was extracted using a Wizard Genomic DNA Purification Kit (Promega) according to the manufacturer’s instructions. The genomic DNA of each isolate was sequenced on a single PacBio P6C4 SMRT Cell (University of Maryland, Baltimore, Maryland, USA), which was assembled using Prokka v1.11 [44] and annotated using the Rapid Annotation using Subsystem Technology (RAST) server [45] and the EggNOG-mapper v1.0.3 using the EggNOG database v4.5.1 [46,47]. Circular genome maps were drawn used CGView software.

### 2.2. Experimental Design

To evaluate the effects of the two bacterial inoculants on the growth of *Sorghum bicolor*, a pot experiment was carried out in a greenhouse over 30 days. Three treatments were applied to pots with autoclaved sandy substrate growing *Sorghum bicolor* cultivar SRN-39, namely, inoculation with *P. tropica* strain IAC/BECa 135, inoculation with *H. frisingense* strain IAC/BECa 152, and no inoculation (control). Each treatment contained six replicate pots.

### 2.3. Plant Growth Conditions

Seeds were disinfected as described by Liu et al. [48]. After disinfection, seeds were placed in Petri dishes with 1% water agar medium and incubated at 25 °C in the dark for two days for seed germination. After the radicles protruded through the seed coats, the seedlings were transplanted to 11 × 11 × 12 cm plastic pots with autoclaved silver sand as the substrate. The pots were maintained under greenhouse conditions for 4 weeks. During the first week, the pots were watered with half-strength Hoagland 10% P nutrient solution [49]; thereafter, P starvation was applied. To create P-starvation conditions, the plant substrate was first flushed using 500 mL of half-strength Hoagland nutrient solution without phosphate to allow any phosphate remaining on the substrate to drain through the pot. After two days, to simulate field conditions, insoluble phosphate (Ca_3_(PO_4_)^2^), which can be solubilized by microorganisms but cannot taken up directly by plants, was diluted in Hoagland nutrient solution and applied to the pots. Twenty-five milliliters of the nutrient solution was applied every two days.

### 2.4. Bacterial Isolation, Growth, and Inoculation

Bacterial isolates were taken from a single colony, grown on Petri dishes containing Luria–Bertani (LB) medium, incubated for 2–3 days at 30 °C, and stored at 4 °C. To prepare the bacterial inoculants, bacterial cultures were grown overnight at 31 °C in LB liquid medium, inoculated again in fresh LB medium, and allowed to grow until they reached the desired inoculum density (10^8^ cfu ml^−1^) [50]. A volume of 1 mL was used for each bacterial strain at each inoculation time. After transplant and during plant growth, the bacterial inoculants were applied three times on top of the sandy substrate, directly onto the roots of the seedlings. The first inoculation occurred on the third day after transplant, the second inoculation was performed on the second day after the initiation of P starvation, and the last bacterial inoculation occurred one week after initiation of P starvation.

### 2.5. Root Architecture and Plant Biomass Assessment

After four weeks, the plants were extracted from the pots, and rhizosphere soils were collected with a sterilized brush, immediately transferred to liquid nitrogen, and stored at −80 °C until DNA extraction. Subsequently, the root systems of the plants were washed with demi water, and the roots and shoots were separated.

For root architecture measurements, the root systems were sectioned into three parts, spread along a rectangular acrylic tray, and placed in an EPSON scanner v.3.9.3 1NL. The measured root architecture parameters were specific root area (SRA, ratio of root surface area to dry mass of roots), specific root length (SRL, ratio of root length to dry mass of roots), average root diameter (AvD), and specific root density (RDENS, ratio of root length to volume). All parameters were analyzed in the program WINRHIZOTM V2005b.

After washing, the shoot and root parts were dried at room temperature for four hours until no remaining water was visible on the surface. The fresh weights of both parts were obtained using an electronic scale. The shoot and root parts were subsequently dried in an oven at 60 °C for 72 h. Biomass was calculated by dividing the dry weight by the fresh weight.

### 2.6. DNA Extraction and Shotgun Sequencing

From the six replicates per treatment, three replicates were randomly chosen for DNA extraction. DNA was extracted from 0.25 g of rhizosphere soil using a DNA Power-Soil DNA isolation kit (Mo Bio Laboratories, Inc., Carlsbad, CA, USA). DNA integrity was checked by agarose gel (1.5%) electrophoresis in 1X Tris–EDTA (TBE) buffer. The gDNA was quantified using a Quant-iTTM dsDNA Assay Kit combined with Gen5 Data Analysis software (BioTek Technology, Winooski, VT, USA). The total DNA was prepared as a MiSeq Illumina paired-end library and sequenced (3 replicates × 3 treatments = 9 metagenomes) using MiSeq technology.

### 2.7. Shotgun Metagenomic Analysis and Quality Control

The bioinformatics steps were performed in a 64-node in-house cluster running Linux Ubuntu v16.04.4 (Xenial Xerus) with 1 TB of memory. The pipeline is available as a Snakemake v4.7.0 pipeline on request from the NIOO-KNAW GitLab. The workflow of the analyses is illustrated in Supplementary Figure S1.

Reads were preprocessed using Trimmomatic v0.36 [51]. Adapter sequences were removed, low-quality reads were removed using a sliding window of 10 bases with a Phred quality score cutoff of 20Q, and reads were trimmed based on a minimum quality threshold of 20Q for leading and trailing bases. A minimum length of 100 base pairs was maintained. Contaminating reads (host and human DNA) were removed using DeconSeq v0.4.3 [52] with a minimum identity and coverage of 90% and 50%, respectively.

### 2.8. Taxonomic and Functional Analyses

After quality control, reads were taxonomically classified using KrakenHLL v0.4 [53], a reference-based classifier built on the Kraken engine [54]. The Kraken database was built using the genomes of plant-associated bacteria from the dataset created by [55]. Unclassified reads were subsequently aligned to a database containing fungal sequences [56]. Genus-level abundance was estimated using the classified reads with Bracken [57]. Default parameters were used for all software in the taxonomic analysis.

Functional profiles of the metagenomes were generated by assembling the reads using MEGAHIT v1.1.2 [58] using the “meta-large” parameter flag. To predict genes, Prodigal v2.6.3 [59] was applied in metagenome mode to the assembled contigs and unassembled reads. Next, the predicted genes were functionally annotated with EggNOG-mapper v1.0.3 using the EggNOG database v4.5.1 [46,47].

The *Paraburkholderia tropica* strain IAC/BECa 135 genome was deposited in National Center for Biotechnology Information (NCBI) with the accession number CP049134, and the *Herbaspirillum frisingense* strain IAC/BECa 152 genome was deposited in NCBI with the accession number CP049139. The metagenome sequences were deposited in the European Nucleotide Archive (ENA; https://www.ebi.ac.uk/ena) under the accession number PRJEB36816 ENA.

### 2.9. Normalization

To compare the taxonomic and functional profiles across treatments, normalization procedures were performed in R v3.4.0. Taxonomic abundances were normalized by multiplying the abundance by a sample-specific weight, which was calculated by dividing the number of matches by the total number of reads. Two normalization techniques were applied to the functional profiles, i.e., average genome size (AGS) [60] and open reading frame (ORF) normalization. For AGS, the average genome sizes of the organisms in the metagenomes were calculated using MicrobeCensus v1.1.0 [61], and functional potential counts were normalized for each metagenome by a weight computed as the average genome size of the metagenome relative to the average genome size over all metagenomes. For ORF normalization, the counts were normalized by a weight calculated per metagenome by dividing the number of annotated genes by the number of predicted genes (ORFs).

### 2.10. Potential Bacterial Interactions

Potential bacterial interactions were determined by mapping the proteins predicted from the metagenomes to KEGG (Kyoto Encyclopedia of Genes and Genomes) pathways that play an important role in the rhizosphere lifestyle (amino sugar and nucleotide sugar metabolism: ko00520; benzoate degradation: ko00362; ABC transporters (ATP-binding cassette transporters): ko02010; carotenoid biosynthesis: ko00906; tryptophan metabolism: ko00380; phosphonate and phosphonate metabolism: ko00440; sulfur metabolism: ko00920; citrate cycle: ko00020; glyoxylate and dicarboxylate metabolism: ko00630; bacterial secretion systems: ko03070; QS: ko02024) using the KEGG Mapper—Reconstruct Pathway utility [62].

### 2.11. PGPR Traits in the P. Tropica and H. frisingense Genomes

#### PGPR Gene Homologues, EPS Gene Clusters, Phytohormone Production, and ABC Transporters

A database containing protein sequences from proteins related to well-studied PGPR mechanisms, including siderophore production, phosphate solubilization, nitrogen fixation, nodulation, plant hormone production, nitrification, and secretion systems, was manually compiled using the public resources UniProt [63] and KEGG [64] (Supplementary Table S1). To evaluate the potential presence or absence of these PGP proteins in the isolates, the ORFs from the *P. tropica* strain IAC/BECa 135 and *H. frisingense* strain IAC/BECa 152 genomes were compared against this database using DIAMOND v0.9.21 [65] (e-value > 1e-102).

Biosynthetic gene clusters encoding enzymes for the synthesis of polysaccharides were identified in the genomes of *H. frisingense* strain IAC/BECa 152 and *P. tropica* strain IAC/BECa 135 using the antiSMASH 3.0 webserver [66]. Matches with possible extracellular polymeric substances (EPS) clusters were further classified into enzymatic families with the dbCAN webserver [67].

To analyze the capacities of *P. tropica* strain IAC/BECa 135 and *H. frisingense* strain IAC/BECa 152 to synthesize phytohormones, the KEGG Mapper—Reconstruct Pathway utility was used to map KEGG Orthology (KO) identifiers of the annotated proteins from the bacterial genomes to the following KEGG pathways: tryptophan metabolism (indole-3-aetate, IAA), phenylalanine metabolism (salicylic acid, SA), cysteine and methionine metabolism (ACC-deaminase and ethylene), alpha-linolenic acid metabolism (jasmonate, JA), zeatin biosynthesis (cytokinin), carotenoid biosynthesis (strigolactones and abscisic acid, ABA), and diterpenoid biosynthesis (gibberellins, GA).

To compare the diversity of ABC transporters, the KO identifiers of the annotated proteins from the genomes of *P. tropica* strain IAC/BECa 135 and *H. frisingense* strain IAC/BECa 152 were mapped to the KEGG ABC transporter pathway (identifier ko02010) using the KEGG Mapper—Reconstruct Pathway utility.

## 3. Results

### 3.1. Bacterial Genome Assembly and Annotation

**Figure 1:**
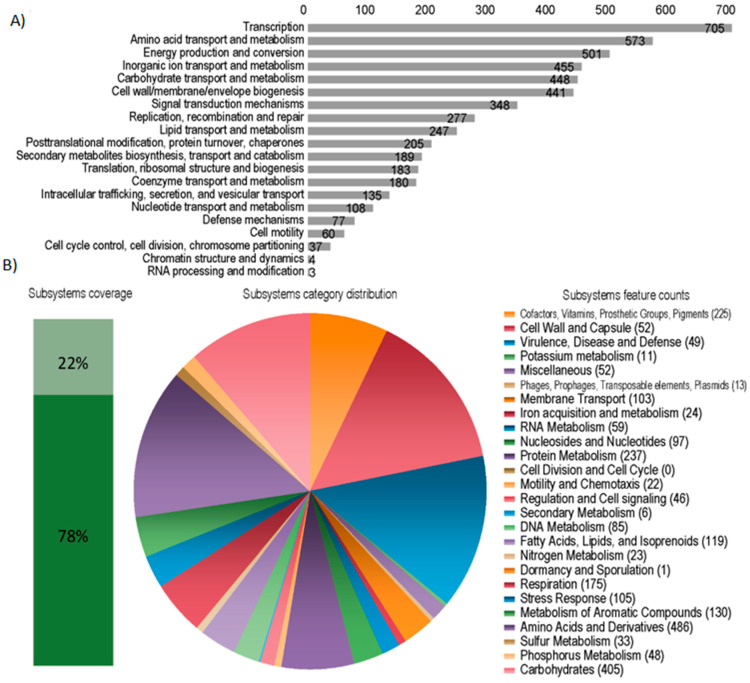
Statistics of the Clusters of Orthologous Groups of proteins (COGs) and Rapid Annotation using Subsystem Technology (RAST) of *Paraburkholderia tropica* strain IAC/BECa 135. (**A**) COG category distribution showing the number of genes annotated in each category. (**B**) Subsystem category distribution. The light green bar represents the percentage of proteins that could be annotated by the RAST server and the dark green bar represents the proteins that were not annotated. The pie chart represents the number of proteins annotated to each subsystem category.

**Figure 2:**
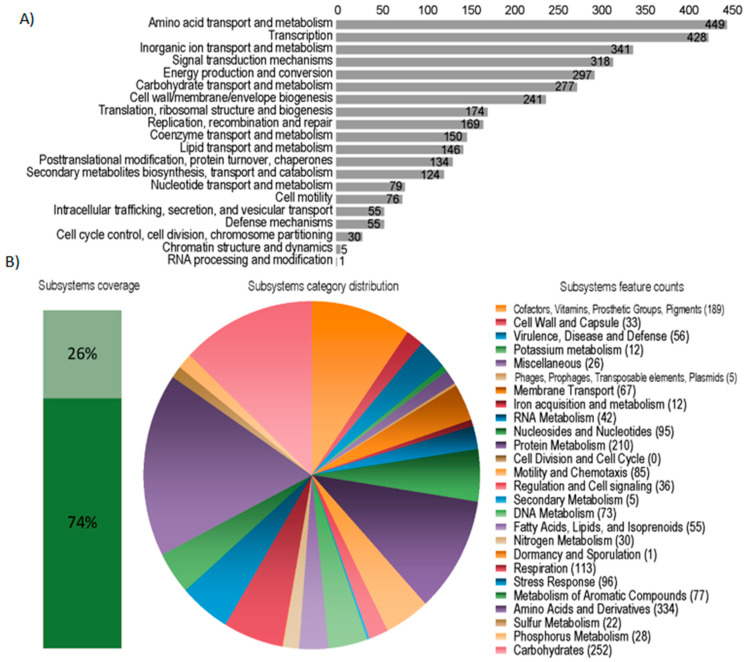
Statistics of the Clusters of Orthologous Groups of proteins (COGs) and Rapid Annotation using Subsystem Technology (RAST) of *Herbaspirillum frisingense* strain IAC/BECa 152. (**A**) COG category distribution showing the number of genes annotated in each category. (**B**) Subsystem category distribution. The light green bar represents the percentage of proteins that could be annotated by the RAST server and the dark green bar represents the proteins that were not annotated. The pie chart represents the number of proteins annotated to each subsystem category.

**Figure 3:**
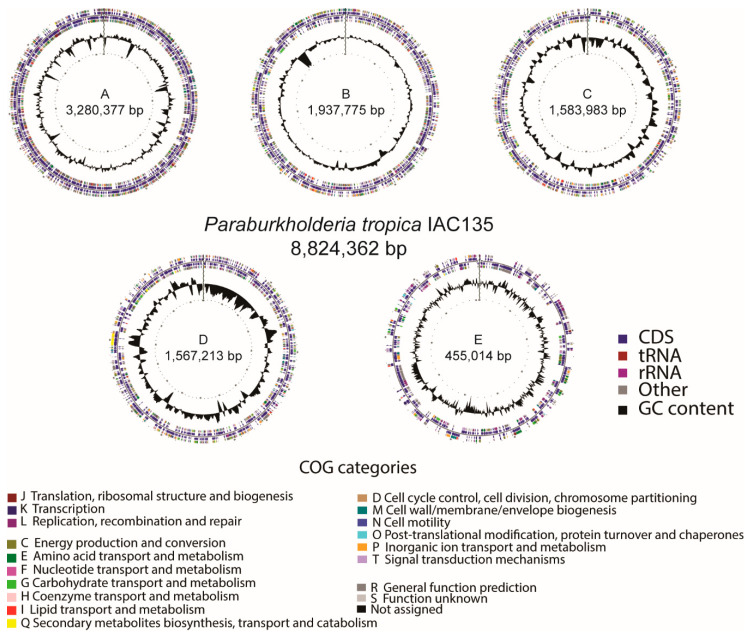
Graphical circular genome map of *Paraburkholderia tropica* strain IAC/BECa 135. The rings indicate coding sequences, Clusters of Orthologous Groups of proteins (COGs) categories, GC content, and GC skew.

**Figure 4:**
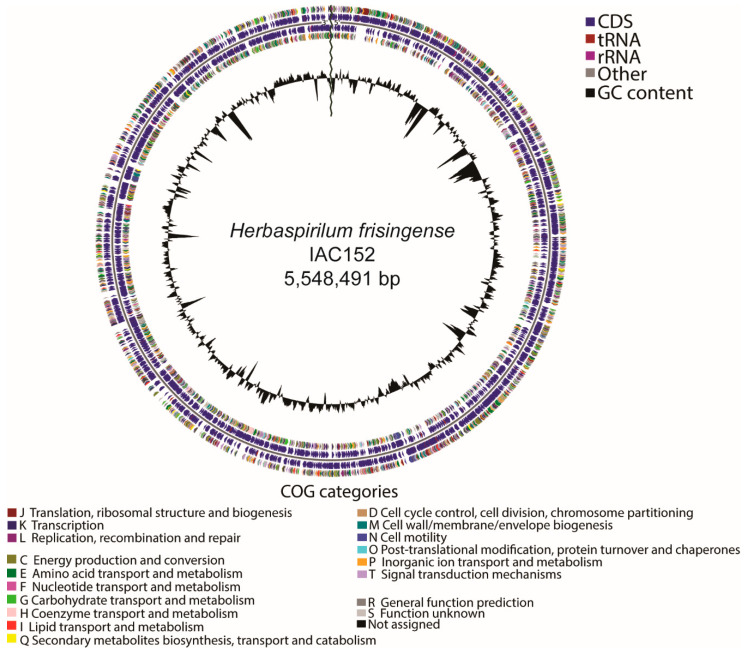
Graphical circular genome map of *Herbaspirillum frisingense* strain IAC/BECa 152. The rings indicate coding sequences, Clusters of Orthologous Groups of proteins (COGs) categories, GC content, and GC skew.

The genomes of the *P. tropica* strain IAC/BECa 135 and *H. frisingense* strain IAC/BECa 152 isolates were assembled and annotated (Table 1). Figure 1A and Figure 2A show the Clutsers of Orthologous Groups of proteins (COG) category distribution and the number of genes annotated in each category of *P. tropica* strain IAC/BECa 135 and *H. frisingense* strain IAC/BECa 152, respectively. The subsystem category distribution of each genome showed that 22% and 26% of proteins *P. tropica* strain IAC/BECa 135 (Figure 1B) and *H. frisingense* strain IAC/BECa 152 (Figure 2B), respectively, could be annotated by the Rapid Annotation using Subsystem Technology (RAST) server. *P. tropica* strain IAC/BECa 135 assembly resulted in one chromosome and four chromids with lengths between 455.01 kb and 3280.34 kb (Figure 3), whereas *H. frisingense* strain IAC/BECa 152 assembly resulted in a single chromosome of 5548.49 kb (Figure 4). These statistics were similar to those for assemblies of different strains in the NCBI genome assembly database.

### 3.2. Plant Biomass and Root Architecture

**Figure 5:**
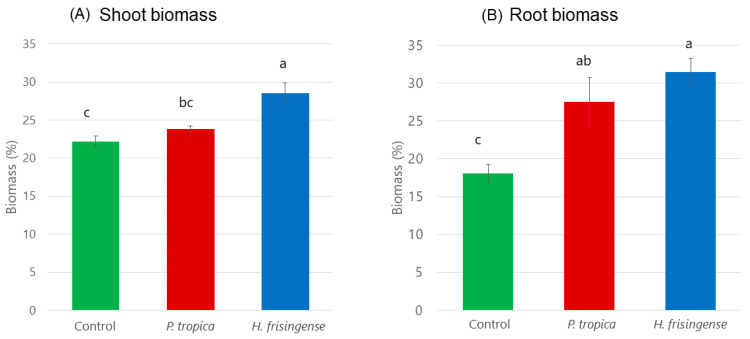
(**A**) Shoot and (**B)** root biomass (%) of sorghum cultivar SRN-39 inoculated with *Paraburkholderia tropica* strain IAC/BECa 135 and *Herbaspirillum frisingense* strain IAC/BECa 152. The values are the means of replicates (*n* = 6) ± (SE). For each parameter, letters in the same column compare means between treatments. Means followed by the same letter are not statistically different according to Duncan’s test (*p* < 0.05).

The effect of *P. tropica* strain IAC/BECa 135 and *H. frisingense* strain IAC/BECa 152, including three other bacterial species on different sorghum cultivars on plant biomass and root architecture, was previously published by Schlemper et al. [39]. Figure 5 illustrates that the sole effect of *P. tropica* strain IAC/BECa 135 and *H. frisingense* strain IAC/BECa 152 significantly increased root and shoot biomass of sorghum cultivar SRN-39. Root biomass increased nearly twofold after inoculation with *H. frisingense* strain IAC/BECa 152, whereas shoot biomass increased by approximately 20%. *P. tropica* strain IAC/BECa 135 showed a weaker but still significant effect on root biomass and a minor effect on shoot biomass. Compared with the control, no differences in sorghum root architecture were observed in the plants inoculated with either of the isolates (Supplementary Table S2).

### 3.3. Metagenomic Sequencing and Read Processing

A total of 29,178,726 raw reads (8542.6 Mb) were generated from nine rhizosphere shotgun metagenomics samples (3 × 3 replicates) of the *Sorghum bicolor* rhizosphere, with a mean length of 292.7 bp. QC trimming and removal of adapter and contamination sequences (sorghum DNA) resulted in 25,807,210 reads (6219.3 Mb) with a mean length of 241 bp (Supplementary Table S3).

### 3.4. Taxonomic Analysis of the Metagenomes

**Figure 6:**
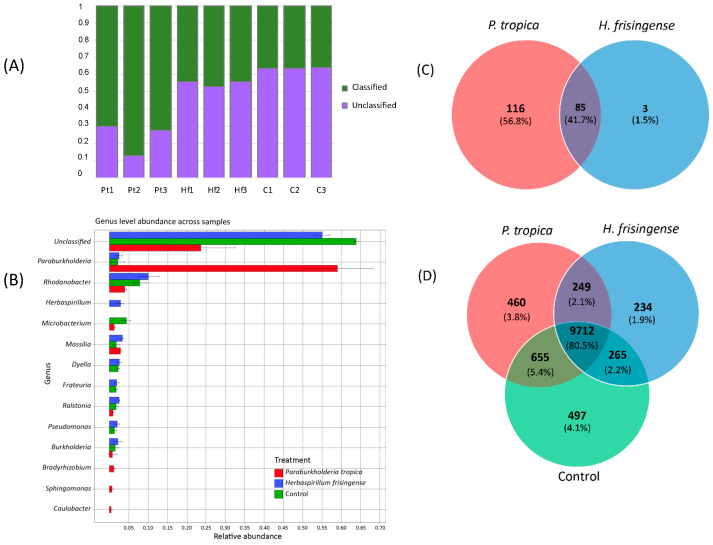
(**A**) Fraction of classified reads per sample. Pt = *Paraburkholderia tropica* strain IAC/BECa 135, Hf = *Herbaspirillum frisingense* strain IAC/BECa 152, C = control. (**B**) Abundance across samples of the 10 most abundant genera per treatment. Each bar shows the relative abundance of a genus per treatment. The error bars indicate the standard deviation across samples from the same treatment. (**C**) Comparison of the presence or absence of plant growth-promoting rhizobacteria (PGPR) genes in the *P. tropica* strain IAC/BECa 135 and *H. frisingense* strain IAC/BECa 152 genomes. (**D**) Comparison of the KEGG Orthology (KO) terms of the metagenomes of the two treatments and the control.

On average, 53% (SD = 0.19) of the reads per sample could be classified as bacteria at the genus level, with a range of 36% to 87% (Figure 6A). The proportion of classified reads were significantly different between the treatments (one-way ANOVA, *p* = 0.000262). The bacterial community differences between the treatments were almost entirely attributable to the inoculants (Figure 6B, Supplementary Figure S2). We observed a striking difference in the abundances of the different inoculants; the relative abundance of *P. tropica* strain IAC/BECa 135 was roughly 20 times higher than that of *H. frisingense* strain IAC/BECa 152. After inoculation, the most abundant genus was *Rhodanobacter*. Surprisingly, 50% of the *Rhodanobacter* abundance consisted of two denitrifying bacteria, namely, *Rhodanobacter denitrificans* and *Rhodanobacter thiooxydans*.

### 3.5. Functional Analysis of the Metagenomes

Although most of the reads from the *P. tropica* strain IAC/BECa 135 inoculant metagenomes were taxonomically classified, the assembly quality of these metagenomes was underwhelming compared to the other metagenomes (Table 2). The metagenome assemblies from the control treatment were slightly better than the assemblies from the *H. frisingense* strain IAC/BECa 152 treatment in terms of assembled contigs, average contig length, and total assembly length. The better assemblies led to higher numbers of predicted genes. Notably, the percentage of complete predicted genes (ORFs including start and stop codons) and average contig size were strongly correlated (Pearson correlation coefficient *r* = 0.62), whereas the correlation between the average contig size and percentage of annotated genes was low (*r* = 0.27), implying that the quality of the metagenome assembly did not strongly influence the annotation process.

The functional profiles of the metagenomes were highly similar at a high level of abstraction. The distribution of COG terms among the metagenomes did not differ by more than 1% between treatments (Supplementary Figure S3). Additionally, the abundances of KO pathways in the metagenomes did not differ significantly. However, the QS and ABC transporter pathways were both slightly more abundant (although not statistically significant) in the *P. tropica* strain IAC/BECa 135 metagenomes.

### 3.6. PGPR Gene Homologues

The *P. tropica* strain IAC/BECa 135 and *H. frisingense* strain IAC/BECa 152 genomes were compared according to the presence or absence of PGPR gene homologues. The presence of genes related to indole-3-acetate (IAA) synthesis, nitrogen fixation, nodulation, siderophore production, and phosphate solubilization in both genomes underlined the genetic capacity of both strains to promote plant growth. PGPR genes are present in both genomes, however, *P. tropica* strain IAC/BECa 135 possesses more genes related to siderophore production, specifically, genes similar to those responsible for pyoverdine synthesis (Supplementary Table S4). Three unique genes related to PGPR traits were observed in the *H. frisingense* strain IAC/BECa 152 genome, while 116 unique PGPR genes were found in the genome of *P. tropica* strain IAC/BECa 135 (Figure 6C, Supplementary Table S4).

### 3.7. EPS Gene Clusters

Respectively, 28 and 71 biosynthetic gene clusters were found in *H. frisingense* strain IAC/BECa 152 and *P. tropica* strain IAC/BECa 135. Compared to the *H. frisingense* strain IAC/BECa 152 gene clusters, a higher proportion of *P. tropica* strain IAC/BECa 135 gene clusters were antibacterial and polysaccharide gene clusters (Supplementary Table S5). The genes from the polysaccharide clusters were classified into carbohydrate active enzyme (CAZy) families, and polysaccharide clusters potentially encoding extracellular polysaccharides were identified based on the presence of a transport protein in the gene cluster (Table 3). The presence of a gene annotated as "polysaccharide export protein" was not sufficient for classification into a transporter family.

### 3.8. Phytohormone Production

The ability to produce phytohormones is important in establishing colonization of the host plant. Both bacterial strains possess the ACC-deaminase coding enzyme, which can degrade the ethylene precursor ACC, however, we cannot confirm their capacity to synthesize IAA without functional analysis. No other enzymes related to phytohormone synthesis were detected in the *P. tropica* strain IAC/BECa 135 and *H. frisingense* strain IAC/BECa 152 genomes.

### 3.9. PGPR Pathway Analysis

The KO (KEGG Orthology) identifiers of the annotated genes from the *P. tropica* strain IAC/BECa 135 and *H. frisingense* strain IAC/BECa 152 genomes were mapped against several KEGG pathways related to PGPR properties. The most notable difference in pathways between the two species was the number of QS systems (Supplementary Figure S4). *P. tropica* strain IAC/BECa 135 possesses six distinct pairs of autoinducer-sensing proteins that are not present in *H. frisingense* strain IAC/BECa 152. By contrast, *H. frisingense* strain IAC/BECa 152 has only one unique autoinducer-sensing protein pair, and seven QS systems are shared between the two genomes. The QS systems LuxR, LasR, RpaR, and RhiR, which are unique to *P. tropica* strain IAC/BECa 135, participate in a positive feedback loop that increases the concentration of the autoinducer; another system identified in *Rhizobium leguminosarum* and present in *P. tropica* strain IAC/BECa 135 was proposed to influence nodulation. Another major difference was found in the bacterial secretion systems (Supplementary Figure S5). The type I and VI secretion systems (T1SS and T6SS) and the general secretion pathway, which are responsible for small molecule export, transfer of molecules to recipient cells, and secretion of unfolded proteins, respectively, are the only systems present in *H. frisingense* strain IAC/BECa 152. By contrast, *P. tropica* strain IAC/BECa 135 possesses T2SS, which is responsible for the secretion of folded proteins, T4SS, which is responsible for the secretion of large molecules into the cytoplasm of recipient cells, T5SS, which is responsible for the secretion of virulence factors, T6SS, the general secretion pathway, and a large part of T3SS, with only the genes required for the assembly of the needle missing. We also examined the prevalence of different ABC transporters, which fulfil multiple important roles in the inter- and intra-kingdom communication required for successful rhizosphere colonization (Supplementary Figure S6). Again, *P. tropica* strain IAC/BECa 135 possesses a more extensive repertoire of transporters than *H. frisingense* strain IAC/BECa 152 (Table 4). The monosaccharide and amino acid transporter categories are important for adaptation to the dynamic rhizosphere conditions, whereas the transporters in the other category are important for the formation of micronutrients and biofilms. Although the differences discussed so far are all related to environmental information processing and cellular community, there are also minor differences in the metabolic categories. For example, *H. frisingense* strain IAC/BECa 152 does not possess the enzyme pyruvate carboxylase, which catalyzes the conversion of pyruvate to oxaloacetate; the absence of this enzyme results in a lower oxaloacetate concentration available for the citric acid cycle. *P. tropica* strain IAC/BECa 135 possesses more enzymes belonging to the metabolic pathways of glyoxylate/dicarboxylate metabolism, arginine biosynthesis, biosynthesis of siderophore group nonribosomal peptides, and phenylpropanoid biosynthesis.

### 3.10. Community Functional Analysis

When the KOs were used as a measure of functionality, the functional capacities of the microbial communities assembled in the rhizosphere by the inoculation of the two strains differed by only a few percent (Figure 6D). The functional profiles of the control and *H. frisingense* strain IAC/BECa 152 treatments were most similar (87.6% shared KOs), consistent with the taxonomic profiles. Between 43% and 49% of the predicted genes were annotated. The unique KOs for each treatment were mapped to KEGG pathways to identify modules present in the metagenome of only one treatment. The KEGG categories with the largest numbers of unique KOs across treatments were metabolism and environmental signal processing. No complete biosynthetic modules unique to a metagenome of a single treatment were found.

## 4. Discussion

This study was the first to analyze and compare the community composition in the *Sorghum bicolor* rhizosphere after inoculation with *P. tropica* strain IAC/BECa 135 and *H. frisingense* strain IAC/BECa 152 under phosphate-deficient conditions. Inoculation of these bacteria in *Sorghum bicolor* cultivar SRN-39 led to significant increases in root and shoot biomass. Although *P. tropica* strain IAC/BECa 135 was much more abundant in the rhizosphere than *H. frisingense* strain IAC/BECa 152, plants treated with *H. frisingense* strain IAC/BECa 152 demonstrated higher root and shoot biomass. However, the current study was carried out in sterilized substrate conditions; in field conditions, the responses of these strains might be different due to expected microbiome competition in rhizosphere.

Previous studies showed growth-promoting effects of *P. tropica* in *Triticum aestivum* (wheat), tomato, and microalgae (*Chlorella* sp.) via nitrogen fixation, phosphate solubilization, and phytohormone production [68,69], but s genome of the strain was not sequenced. We confirmed the presence of the genes required for these mechanisms in our genome analysis of the sequenced genome of *P. tropica* strain IAC/BECa 135.

Growth promotion by *H. frisingense* was demonstrated in sugarcane [38], sorghum [39], and *Miscanthus sinensis* (both C4 grass species). However, in *M. sinensis*, the effect was due not to increased nutrient levels but to an influence of this endophytic bacterium on plant hormone signaling [70]. A study comparing the genomes of *Herbaspirillum* species confirmed the presence of many genes involved in plant hormone synthesis in the *H. frisingense* genome [71]. In this study, the endophytic bacterial community was not analyzed. Therefore, it is possible that high endophytic concentrations of *H. frisingense* strain IAC/BECa 152 were present, as previous research showed that the mode of action of *H. frisingense* primarily involves the intercellular apoplastic space. Genomic analysis of *H. frisingense* strain IAC/BECa 152 showed the presence of genes involved in IAA synthesis and ACC-deaminase, as well as genes involved in direct PGPR traits such as nitrogen fixation and phosphate solubilization; however, the activity of these genes could not be quantified, as the transcriptome was not measured. Despite the lack of phytohormone synthesis genes, endophytic microbes were shown to influence hormone signaling by synthesizing hormone mimics and effector proteins that influence hormone production in planta [72]. This aspect of endophytic hormone regulation was not explored, and it is highly possible that similar systems exist in *H. frisingense* strain IAC/BECa 152 but were not detected by the homology-based approach used in this study. In addition, the presence of genes involved in terpenes and other antimicrobial compounds could be involved in plant defenses against bacterial, fungal, and viral pathogens [71].

The *P. tropica* strain IAC/BECa 135 genome is enriched in ABC transporters, biosynthetic clusters encoding enzymes involved in polysaccharide synthesis, QS systems, and secretion systems compared to the *H. frisingense* strain IAC/BECa 135 genome. These genetic features are involved in the colonization process and could thus explain the extensive rhizosphere colonization by *P. tropica* strain IAC/BECa 135. Another plant-beneficial *Burkholderia* strain, *Burkholderia* australis (arguably, this strain could be reclassified as *Paraburkholderia* based on its plant growth-promoting properties), was shown to form a biofilm on sugarcane (a close relative of sorghum) roots, in which the expression of EPS genes, cytochrome genes, and energy pathway genes were upregulated, lipopolysaccharide (LPS) and flagella biosynthesis (which are often recognized as virulence factors by plants) gene expression were downregulated, and additional energy pathways were activated [73]. Interestingly, sugarcane root extract can induce biofilm formation by *B. australis*, whereas *P. tropica* strain IAC/BECa 135 root colonization appears to be dependent on the orobanchol concentration in *Sorghum bicolor* root exudates [39], indicating that biofilm formation is important for successful colonization of the rhizosphere. Biofilm formation, in turn, is influenced by QS. The extensive repertoire of distinct QS systems in *P. tropica* strain IAC/BECa 135 could enable the bacterium to integrate many signals and coordinate gene expression accordingly. Furthermore, the products of variable biosynthetic gene clusters can play a role in biocontrol activity against phytophatogens, such as fungi, bacteria, and nematodes, and induce plant biomass increase, as demonstrated for other *Burkholderia* and *Paraburkholderia* species. In addition, the large number of distinct ABC transporters may enable *P. tropica* strain IAC/BECa 135 to sense many environmental stimuli, thus conferring a colonization advantage. The genomic differences between *P. tropica* strain IAC/BECa 135 and *H. frisingense* strain IAC/BECa 152 may reflect the differences between the rhizospheric and endophytic lifestyles, and further studies on gene expression may confirm the endophytic colonization of *S. bicolor* by *H. frisingense* strain IAC/BECa 152.

## 5. Conclusions

**Figure 7:**
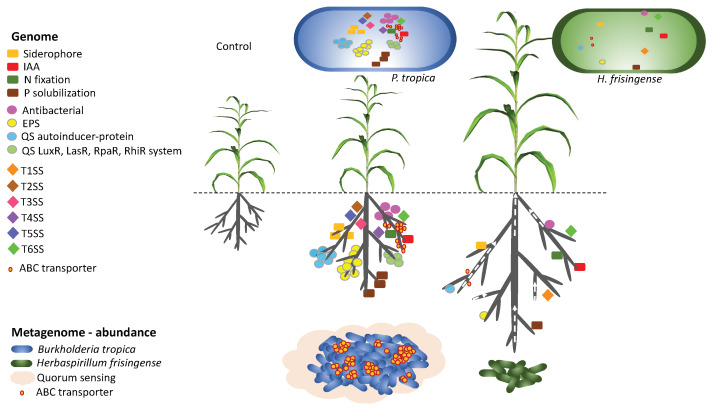
Concept framework of *Paraburkholderia tropica* strain IAC/BECa 135 and *Herbaspirillum frisingense* strain IAC/BECa 152 colonizing the rhizosphere of *Sorghum bicolor* cultivar SRN-39 based on comparative genomics and metagenomics. IAA: indol acetic acid; EPS: exoplolysaccharide; QS: quorum sensing; T1SS, T2SS, T3SS, T4SS, T5SS, and T6SS: types I, II, III, IV, V, and VI secretion systems, respectively.

In summary, as illustrated in Figure 7, *P. tropica* strain IAC/BECa 135 was more successful in colonizing the rhizosphere of *Sorghum bicolor* cultivar SRN-39 than *H. frisingense* strain IAC/BECa 152. This advantage was partially explained by the *P. tropica* strain IAC/BECa 135 genome, which is enriched in features important for colonization. As inoculation with *H. frisingense* strain IAC/BECa 152 led to a greater increase in biomass than inoculation with *P. tropica* strain IAC/BECa 135 despite a lower degree of rhizosphere colonization, it is possible that a high concentration of *H. frisingense* strain IAC/BECa 152 was present in the intercellular apoplastic space in the roots. The mechanisms by which *P. tropica* strain IAC/BECa 135 and *H. frisingense* strain IAC/BECa 152 promote plant growth might be complementary, i.e., *H. frisingense* strain IAC/BECa 152 seems to influence plant hormone regulation, whereas *P. tropica* strain IAC/BECa 135 comprehensively colonizes the root system and surrounds the roots with biofilm, protecting the roots against pathogens and increasing nutrient availability through a range of mechanisms. While the functional and taxonomic profiles of the rhizobiomes of all treatments were similar, significant differences in biomass were observed, indicating that the rhizobiome and endophytic microbial community may play equally important roles in the complicated plant–microbial interplay underlying the increased growth of the host plant.

Finally, as *P. tropica* strain IAC/BECa 135 and *H. frisingense* strain IAC/BECa 152 presumably promote growth through different mechanisms, co-inoculation of these bacteria should lead to greater increases in root and shoot biomass than inoculation of a single bacterium. This hypothesis could be tested by performing an experiment similar to this study, in which *P. tropica* strain IAC/BECa 135 and *H. frisingense* strain IAC/BECa 152 are inoculated at different concentrations to find an optimal inoculum.

## Figures and Tables

**Table 1:** Summary of isolate genome assembly, gene prediction, and annotation.

	*P. tropica* IAC/BECa 135	*H. frisingense* IAC/BECa 152
Total length (Mb)	8.82	5.55
Number of chromosomes (chromids)	1 (4)	1
Number of predicted genes	7711	5009
Number of annotated genes	6929	4682
Fraction annotated	0.89	0.93

**Table 2:** Summary of metagenome assembly, gene prediction, and annotation.

	Pt-1	Pt-2	Pt-2	Hf-1	Hf-2	Hf-3	C-1	C-2	C-3
Assembled contigs	222,198	133,137	208,350	288,324	258,768	291,941	323,818	303,981	307,497
Average length (bp)	618	633	608	692	650	661	660	645	639
Total length (Mb)	137.3	84.2	126.6	199.5	168.2	192.8	213.9	196.0	196.6
Predicted genes	565,399	400,139	539,756	825,928	719,776	832,996	849,382	806,712	822,576
Fraction complete	0.06	0.06	0.06	0.07	0.06	0.06	0.07	0.07	0.06
Annotated genes	244,718	194,797	232,699	372,477	323,061	378,455	387,321	347,391	352,764
Fraction annotated	0.43	0.49	0.43	0.45	0.45	0.45	0.46	0.43	0.43

Pt: *P. tropica* strain IAC/BECa 135; Hf: *H. frisingense* strain IAC/BECa 152; C: control.

**Table 3:** Carbohydrate enzyme families and export proteins in biosynthetic gene clusters.

Cluster	Export Protein	CAZY Families Present	Homologous Clusters
Pt-C-1	MATE efflux protein, outer membrane polysaccharide export protein	GH39, GT4, GT2	Colanic acid
Pt-C-2	ABC transporter protein	GT2, GT4	None
Pt-C-3	ABC transporter protein	GT21, GT5	None
Pt-C-4	MATE efflux protein	None found	Cepacian
Pt-C-5	RND efflux protein	GT1, GT2, CE14, GT2	None
Pt-C-6	Polysaccharide export protein	GT2, GT4	Cepacian
Pt-C-7	Polysaccharide export protein	GT2, GT4, GH5	O-antigen
Hf-C-1	Polysaccharide export protein	GT2, GT4, GT9, GT11, GT28, GT30, GH109	O-antigen
Hf-C-2	Polysaccharide export protein	GT2, GT4, GT26	None

Pt-C: *P. tropica* strain IAC/BECa 135 cluster; Hf-C: *H. frisingense* strain IAC/BECa 152 cluster; MATE: Multi-antimicrobial extrusion protein; ABC: ATP-binding cassette transporter; RND: resistance nodulation division of efflux pumps; GT: glycosyl transferase; GH: glycosyl hydrolase; CE: carbohydrate esterase.

**Table 4:** Presence of ABC transporters in the *P. tropica* IAC/BECa 135 and *H. frisingense* IAC/BECa 152 strain genomes.

*Monosaccharide Transporters*	IAC/BECa 135	IAC/BECa 152
Glucose/Arabinose	-	-
Glucose/Manose	+	+
Ribose/D-xylose	+	+
L-arabinose	+	-
Methyl-galactoside	-	-
D-xylose	+	+
D-allose	-	-
Fructose	+	-
Rhamnose	+	-
Erythritol	+	-
Xylitol	+	-
Myo-inositol	+	+
Myo-inositol 1-phosphate	-	-
Glycerol	-	+
Sn-glycerol 3-phosphate	-	+
Total	9	6
*Phosphate and amino acid transporters*		
Phosphate	+	+
Phosphonate	+	+
Lysine/Arginine/Omithine	+	-
Histidine	+	-
Glutamine	-	-
Arginine	-	-
Glutamate/Aspartate	+	+
Octopine/Nopaline	+	-
General L-amino acid	+	+
Glutamate	-	-
Cystine	+	+
Arginine/Omithine	+	-
Lysine	-	-
Branched amino acid	+	+
Neutral amino acid	-	-
Urea	+	+
D-methionine	-	-
Total	11	7
*Other transporters*		
Oligopeptide	+	-
Gluthathione	-	+
Iron complex	+	+
Lipopolysaccharide	+	-
Lipo-oligosaccharide	+	-
Total	4	2

+, present in the genome; -, not or partially present in the genome.

## Supplementary Materials

The following are available online at https://www.mdpi.com/2076-2607/8/5/725/s1, Supplementary Figure S1: Bioinformatics workflow analysis; Supplementary Table S1: Category, subcategory, gene name, number of sequences of PGPR traits siderophore production, phosphate solubilization, nitrogen fixation, nodulation, plant hormone production, nitrification, and secretion systems manually compiled using the public resources UniProt (Chen et al. 2017) and KEGG (Kanehisa et al. 2013); Supplementary Table S2: Specific root area (SRA), specific root length (SRL), average of root diameter (AvD), and specific root density (RDENS) of sorghum cultivar SRN-39 inoculated with *P. tropica* IAC/BECa 135 and *H. frisingense* IAC/BECa 152 strains; Table S3: Summary of raw sequences processed and QC filtering from the metagenome samples; Supplementary Figure S2: PCA analysis of the taxonomic classification at species level. (A) PCA plot of the species abundance over all samples. (B) Species contribution to PCA1. (C) Species contribution to PCA2. Analysis was performed using the standard prcomp function in R; Supplementary Figure S3: Abundance of COG terms per sample inoculated with Pt: *P. tropica* IAC/BECa 135 strain and Hf: *H. frisingense* IAC/BECa 152 strain and C—control. 1, 2, 3 are replicates. The value in each bar depicts the abundance of the COG in the metagenome. Definition of the COG categories are A: RNA processing and modification; B: chromatin structure and dynamics; C: energy production and conversion; D: cell cycle control and mitosis; E: amino acid metabolism and transport; F: nucleotide metabolism and transport; G: carbohydrate metabolism and transport; H: coenzyme metabolism; I: lipid metabolism; J: translation; K: transcription; L: replication and repair; M: cell wall/membrane/envelop biogenesis; N: cell motility; O: post-translational modification, protein turnover, chaperone functions; P: inorganic ion transport and metabolism; Q: secondary structure; T: signal transduction; U: intracellular trafficking and secretion; V: defense mechanisms; Y: nuclear structure; Z: cytoskeleton; R: general functional prediction only; S: function unknown; Supplementary Table S4: Number of PGPR gene homologues identified in the genomes of *P. tropica* strain IAC/BECa 135 and *H. frisingense* strain IAC/BECa 152 using a manually compiled PGPR database (e-value > 1e-102); Supplementary Table S5: Number of specific identified gene clusters; Supplementary Figure S4: Quorum sensing KEGG. The enzymes in the pathways are colored based on presence in the genomes, where red means the enzyme is present in the *P. tropica* IAC/BECa 135 genome and green means the enzyme is present in the *H. frisingense* strain IAC/BECa 152 genome; Supplementary Figure S5: Bacterial secretion systems KEGG. The enzymes in the pathways are colored based on presence in the genomes, where red means the enzyme is present in the *P. tropica* IAC/BECa 135 genome and green means the enzyme is present in the *H. frisingense* strain IAC/BECa 152 genome; Figure S6: ABC transporters KEGG. The enzymes in the pathways are colored based on presence in the genomes, where red means the enzyme is present in the *P. tropica* IAC/BECa 135 genome and green means the enzyme is present in the *H. frisingense* strain IAC/BECa 152 genome.
