# Supplementary material for: Sorghum Growth Promotion by Paraburkholderia tropica and Herbaspirillum frisingense: Putative Mechanisms Revealed by Genomics and Metagenomics
Source: Microorganisms. 2020 May 13;8(5):725. doi: 10.3390/microorganisms8050725 (PMC7285511; doi:10.3390/microorganisms8050725)
Supplement: Supplementary file 1 [file microorganisms-08-00725-s001.pdf]

Sorghum Growth Promotion by *Paraburkholderia tropica* and *Herbaspirillum frisingense*: Putative Mechanisms Revealed by Genomics and Metagenomics

Eiko E. Kuramae [1,*], Stan Derksen [1], Thiago R. Schlemper [1], Maurício R. Dimitrov [1], Ohana Y.A. Costa [1] and Adriana P. D. da Silveira [2,*]

1 Netherlands Institute of Ecology (NIOO-KNAW), Microbial Ecology Department, Droevendaalsesteeg 10, 6708 PB Wageningen, The Netherlands
2 Center of Soil and Environmental Resources, Agronomic Institute of Campinas (IAC), Av. Barão de Itapura 1481, 13020-902 Campinas, SP, Brazil
* Correspondence: E.Kuramae@nioo.knaw.nl (E.E.K.), apdsil@iac.sp.gov.br (A.P.D.d.S.)

Supplementary Material

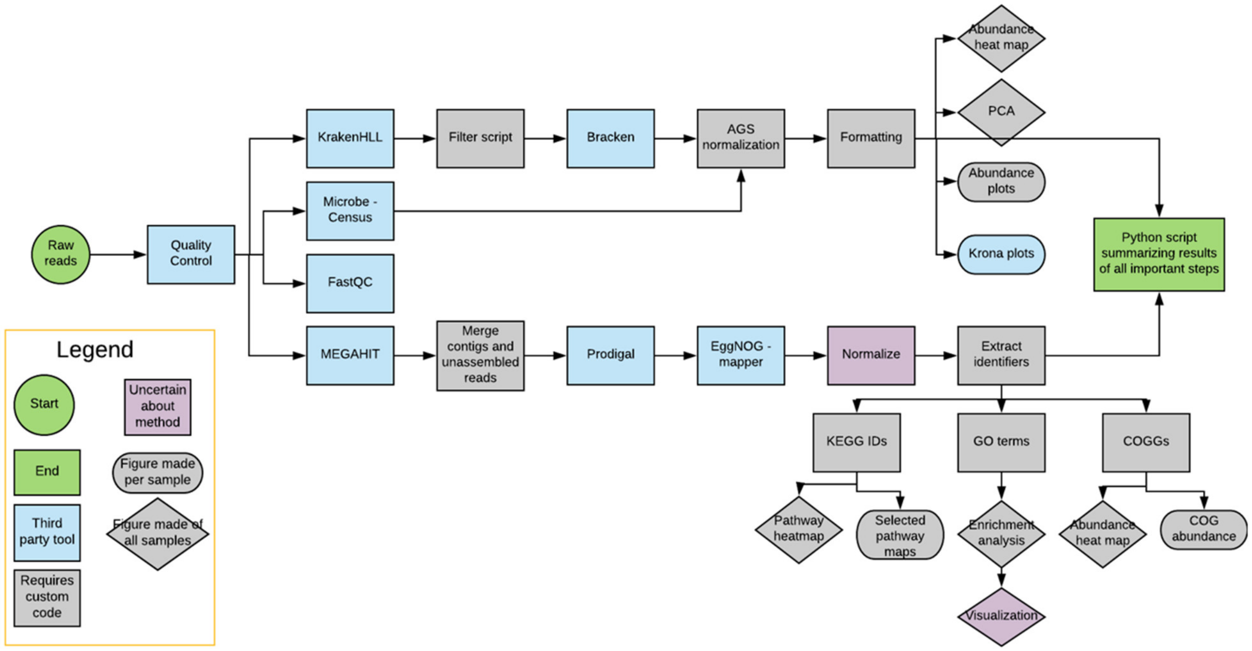

Figure S1. Bioinformatics workflow analysis.

Table S1. Category, subcategory, gene name, number of sequences of PGPR traits, siderophore production, phosphate solubilization, nitrogen fixation, nodulation, plant hormone production, nitrification, and secretion systems manually compiled using the public resources UniProt (Chen et al., 2017) and KEGG [1].

Category	Subcategory	SubSubcategory	Gene	Number of sequences	Date
Hormones	IAA	Synthesis	aam	242	20/04/2018
Hormones	ACC Deaminase	Synthesis	acdS	7163	20/04/2018
Hormones	IAA	Synthesis	aldH	571	20/04/2018
Siderophore	Transport	Desferrioxamine	BfrH	31	19/04/2018
Siderophore	Transport	Desferrioxamine	DesA	798	19/04/2018
Siderophore	Synthesis	Desferrioxamine	DesB	21	19/04/2018
Siderophore	Synthesis	Desferrioxamine	DesC	241	19/04/2018
Siderophore	Synthesis	Desferrioxamine	DesD	37	19/04/2018
Siderophore	Synthesis	Enterobactin	EntA	365	19/04/2018
Siderophore	Synthesis	Enterobactin	EntB	494	19/04/2018
Siderophore	Synthesis	Enterobactin	EntC	448	19/04/2018
Siderophore	Synthesis	Enterobactin	EntD	182	19/04/2018
Siderophore	Synthesis	Enterobactin	EntE	1379	19/04/2018
Siderophore	Synthesis	Enterobactin	EntF	958	19/04/2018
Siderophore	Transport	Enterobactin	EntS	840	19/04/2018
Nutrients	Nitrogen fixation	Synthesis	fdxN	96	19/04/2018
Siderophore	Transport	Ferrichrome	FecA	842	19/04/2018
Siderophore	Transport	Ferrichrome	FecB	572	19/04/2018
Siderophore	Transport	Ferrichrome	fecC	326	19/04/2018
Siderophore	Transport	Ferrichrome	FecD	1010	19/04/2018
Siderophore	Transport	Ferrichrome	FecE	1743	19/04/2018
Siderophore	Transport	Enterobactin	FepA	635	19/04/2018
Siderophore	Transport	Enterobactin	FepB	366	19/04/2018
Siderophore	Transport	Enterobactin	FepC	380	19/04/2018
Siderophore	Transport	Enterobactin	fepD	518	19/04/2018
Siderophore	Transport	Enterobactin	FepE	232	19/04/2018
Siderophore	Transport	Enterobactin	FepG	443	19/04/2018
Adaptation	T6SS	Synthesis	Fha	28	20/04/2018
Siderophore	Transport	Enterobactin	FhuA	1228	19/04/2018
Siderophore	Transport	Ferrichrome	FhuB	781	19/04/2018
Siderophore	Transport	Ferrichrome	FhuC	1405	19/04/2018
Siderophore	Transport	Ferrichrome	FhuD	713	19/04/2018
Siderophore	Transport	Desferrioxamine	FhuE	336	19/04/2018
Siderophore	Transport	Desferrioxamine	FoxA	166	19/04/2018
Siderophore	Transport	Desferrioxamine	FoxR	12	19/04/2018
Siderophore	Transport	Pyochelin	FptA	150	19/04/2018

Siderophore	Transport	Pyoverdine	FpvA	341	19/04/2018
Siderophore	Transport	Pyoverdine	FpvB	15	19/04/2018
Siderophore	Regulation	Pyoverdine	FpvI	19	19/04/2018
Siderophore	Regulation	Pyoverdine	FpvR	17	19/04/2018
Siderophore	Regulation	x	fur	13450	19/04/2018
Nutrients	Phosphate Solubilization	Synthesis	gcd	596	19/04/2018
Nutrients	Nitrification	Synthesis	haoA	3	19/04/2018
Nutrients	Nitrification	Synthesis	HaoB	8	19/04/2018
Hormones	IAA	Synthesis	iaaH	158	20/04/2018
Hormones	IAA	Conjugation	iaaL	27	20/04/2018
Hormones	IAA	Synthesis	iaaM	101	20/04/2018
Hormones	IAA	Synthesis	ipdC	328	20/04/2018
Nutrients	Nitrification	Synthesis	narG	3881	19/04/2018
Nutrients	Nitrification	Synthesis	narH	2466	19/04/2018
Nutrients	Nitrogen fixation	Nodulation	nfe	5	19/04/2018
Nutrients	Nitrogen fixation	Regulatory	nifA	677	19/04/2018
Nutrients	Nitrogen fixation	Synthesis	nifB	486	19/04/2018
Nutrients	Nitrogen fixation	Synthesis	nifC	7	19/04/2018
Nutrients	Nitrogen fixation	Synthesis	nifD	2595	19/04/2018
Nutrients	Nitrogen fixation	Synthesis	nifE	325	19/04/2018
Nutrients	Nitrogen fixation	Synthesis	nifF	47	19/04/2018
Nutrients	Nitrogen fixation	Synthesis	nifH	35367	19/04/2018
Nutrients	Nitrogen fixation	Regulatory	nifI	16	19/04/2018
Nutrients	Nitrogen fixation	Synthesis	nifJ	1597	19/04/2018
Nutrients	Nitrogen fixation	Synthesis	nifK	819	19/04/2018
Nutrients	Nitrogen fixation	Regulatory	nifL	94	19/04/2018
Nutrients	Nitrogen fixation	Synthesis	nifM	67	19/04/2018
Nutrients	Nitrogen fixation	Synthesis	nifN	247	19/04/2018
Nutrients	Nitrogen fixation	Stabilization	nifO	6	19/04/2018
Nutrients	Nitrogen fixation	Synthesis	nifQ	125	19/04/2018
Nutrients	Nitrogen fixation	Regulatory	nifR	56	19/04/2018
Nutrients	Nitrogen fixation	Synthesis	nifS	1755	19/04/2018
Nutrients	Nitrogen fixation	Unknown	nifT	152	19/04/2018
Nutrients	Nitrogen fixation	Transport	nifU	1411	19/04/2018
Nutrients	Nitrogen fixation	Synthesis	nifV	251	19/04/2018
Nutrients	Nitrogen fixation	Stabilization	nifW	731	19/04/2018
Nutrients	Nitrogen fixation	Synthesis	nifx	270	19/04/2018
Nutrients	Nitrogen fixation	Synthesis	nifY	30	19/04/2018
Nutrients	Nitrogen fixation	Unknown	nifZ	142	19/04/2018
Nutrients	Nitrogen fixation	Nodulation	nodA	3006	19/04/2018
Nutrients	Nitrogen fixation	Nodulation	nodB	532	19/04/2018

Nutrients	Nitrogen fixation	Nodulation	nodC	4551	19/04/2018
Nutrients	Nitrogen fixation	Nodulation	nodD	1258	19/04/2018
Siderophore	Synthesis	x	PchA	113	19/04/2018
Siderophore	Synthesis	Pyochelin	PchB	127	19/04/2018
Siderophore	Synthesis	Pyochelin	PchD	20	19/04/2018
Siderophore	Synthesis	Pyochelin	PchE	21	19/04/2018
Siderophore	Synthesis	Pyochelin	PchF	80	19/04/2018
Siderophore	Synthesis	Pyochelin	PchG	21	19/04/2018
Siderophore	Regulation	Pyochelin	PchR	214	19/04/2018
Nutrients	Phosphate Solubilization	Synthesis	PhoA	879	19/04/2018
Nutrients	Phosphate Solubilization	Regulatory	PhoB	3059	19/04/2018
Nutrients	Phosphate Solubilization	Transport	PhoC	171	19/04/2018
Nutrients	Phosphate Solubilization	Synthesis	PhoD	1253	19/04/2018
Nutrients	Phosphate Solubilization	Transport	PhoE	321	19/04/2018
Nutrients	Phosphate Solubilization	Unknown	PhoH	992	19/04/2018
Nutrients	Phosphate Solubilization	Synthesis	PhoK	61	19/04/2018
Nutrients	Phosphate Solubilization	Unknown	PhoL	65	19/04/2018
Nutrients	Phosphate Solubilization	Synthesis	PhoN	53	19/04/2018
Nutrients	Phosphate Solubilization	Regulatory	PhoR	1435	19/04/2018
Nutrients	Phosphate Solubilization	Transport	PhoS	91	19/04/2018
Nutrients	Phosphate Solubilization	Transport	PhoT	79	19/04/2018
Nutrients	Phosphate Solubilization	Transport	PhoU	4454	19/04/2018
Nutrients	Phosphate Solubilization	Synthesis	PhoV	30	19/04/2018
Nutrients	Phosphate Solubilization	Transport	PhoW	6	19/04/2018
Nutrients	Phosphate Solubilization	Transport	PhoX	1902	19/04/2018
Nutrients	Phosphate Solubilization	Transport	PhoY	47	19/04/2018
Nutrients	Phosphate Solubilization	Transport	PhoZ	8	19/04/2018
Nutrients	Nitrification	Synthesis	pmoA	12269	19/04/2018
Nutrients	Nitrification	Synthesis	pmoC	123	19/04/2018
Adaptation	T6SS	Regulatory	PpkA	55	20/04/2018
Nutrients	Phosphate Solubilization	Synthesis	PqqA	1151	19/04/2018
Nutrients	Phosphate Solubilization	Synthesis	PqqB	2890	19/04/2018
Nutrients	Phosphate Solubilization	Synthesis	PqqC	3174	19/04/2018
Nutrients	Phosphate Solubilization	Synthesis	PqqD	1865	19/04/2018
Nutrients	Phosphate Solubilization	Synthesis	PqqE	3163	19/04/2018
Nutrients	Phosphate Solubilization	Synthesis	PqqF	288	19/04/2018
Nutrients	Phosphate Solubilization	Regulatory	PqqH	11	19/04/2018
Hormones	IAA	Synthesis	prr	923	20/04/2018
Nutrients	Phosphate Solubilization	Transport	pstA	5152	19/04/2018
Nutrients	Phosphate Solubilization	Transport	PstB	19975	19/04/2018
Nutrients	Phosphate Solubilization	Transport	PstC	5607	19/04/2018

Nutrients	Phosphate Solubilization	Transport	PstD	22	19/04/2018
Nutrients	Phosphate Solubilization	Transport	PstE	13	19/04/2018
Hormones	IAA	Synthesis	puuC	595	20/04/2018
Siderophore	Synthesis	Pyoverdine	PvcA	39	19/04/2018
Siderophore	Synthesis	Pyoverdine	PvcB	54	19/04/2018
Siderophore	Synthesis	Pyoverdine	PvcC	19	19/04/2018
Siderophore	Synthesis	Pyoverdine	PvcD	3	19/04/2018
Siderophore	Synthesis	Pyoverdine	PvdA	161	19/04/2018
Siderophore	Synthesis	Pyoverdine	PvdD	18	19/04/2018
Siderophore	Transport	Pyoverdine	PvdE	44	19/04/2018
Siderophore	Synthesis	Pyoverdine	PvdF	18	19/04/2018
Siderophore	Synthesis	Pyoverdine	PvdI	21	19/04/2018
Siderophore	Synthesis	Pyoverdine	PvdJ	24	19/04/2018
Siderophore	Regulation	Pyoverdine	PvdS	67	19/04/2018
Adaptation	T6SS	Effector	Rhs2	5	20/04/2018
Adaptation	T6SS	Effector	RhsA	60	20/04/2018
Adaptation	T6SS	Effector	RhsB	33	20/04/2018
Adaptation	T6SS	Effector	Tae	131	20/04/2018
Adaptation	T6SS	Synthesis	TagJ	7	20/04/2018
Adaptation	T6SS	Synthesis	TssA	651	20/04/2018
Adaptation	T6SS	Synthesis	TssB	47	20/04/2018
Adaptation	T6SS	Synthesis	TssC	144	20/04/2018
Adaptation	T6SS	Synthesis	TssD	24	20/04/2018
Adaptation	T6SS	Synthesis	TssE	597	20/04/2018
Adaptation	T6SS	Synthesis	TssF	939	20/04/2018
Adaptation	T6SS	Synthesis	TssG	797	20/04/2018
Adaptation	T6SS	Synthesis	TssH	996	20/04/2018
Adaptation	T6SS	Synthesis	TssI	37	20/04/2018
Adaptation	T6SS	Synthesis	TssJ	579	20/04/2018
Adaptation	T6SS	Synthesis	TssK	770	20/04/2018
Adaptation	T6SS	Synthesis	TssL	325	20/04/2018
Adaptation	T6SS	Synthesis	TssM	550	20/04/2018
Adaptation	T6SS	Synthesis	TssN	3	20/04/2018
Adaptation	T6SS	Effector	Uniprot T6SS Effectors	2079	20/04/2018

Table S2. Specific root area (SRA), specific root length (SRL), average of root diameter (AvD), and specific root density (RDENS) of *Sorghum* cultivar SRN-39 inoculated with *P. tropica* IAC/BECa 135 strain and *H. frisingense* IAC/BECa 152 strain extracted from Schlemper et al. [39].

Strain	SRA (cm^2/g)	SRL (cm/g)	AvD (mm)	RDENS (cm^3/g)
Control	847.29 ± 44.46 a	687.39 ± 66.85 a	0.40 ± 0.02 a	0.12 ± 0.00 a
IAC/BECa 135	1016.59 ± 59.11a	882.89 ± 91.54 a	0.38 ± 0.02 a	0.11 ± 0.01 a
IAC/BECa 152	914.74 ± 50.78 a	802.62 ± 69.77 a	0.37 ± 0.01 a	0.12 ± 0.00 a

Values are means of replicates (n = 6) ± (SE). For each parameter, letters compare (in a column) the means between the bacterial inoculum treatments. Means followed by the same letter are not statistically different according to Duncan's test ($P < 0.05$).

Table S3. Summary of raw sequences processed and QC filtering from the metagenome samples.

	Pt-1	Pt-2	Pt-3	Hf-1	Hf-2	Hf-3	C-1	C-2	C-3
Initial reads	3,438,848	3,163,810	3,193,606	3,496,096	3,069,472	3,316,080	3,152,220	3,170,522	3,178,072
Total (Mb)	1,008	926	935.7	1,024.3	900.7	972.9	922.9	923.9	928.2
Average length (bp)	293.1	292.7	293	293	293	293.4	292.8	291.4	292.1
Post QC reads	3,090,758	2,910,710	2,859,224	2,872,270	2,544,130	2,861,010	2,869,590	2,917,002	2,882,516
Post QC total (Mb)	741.8	695.7	686.2	692.2	613.1	692.4	694.4	703	700.5
Post QC average length (bp)	240	239	240	241	241	242	242	241	243

Pt: *P. tropica* IAC/BECa 135 strain; Hf: *H. frisingense* IAC/BECa 152 strain; C: control

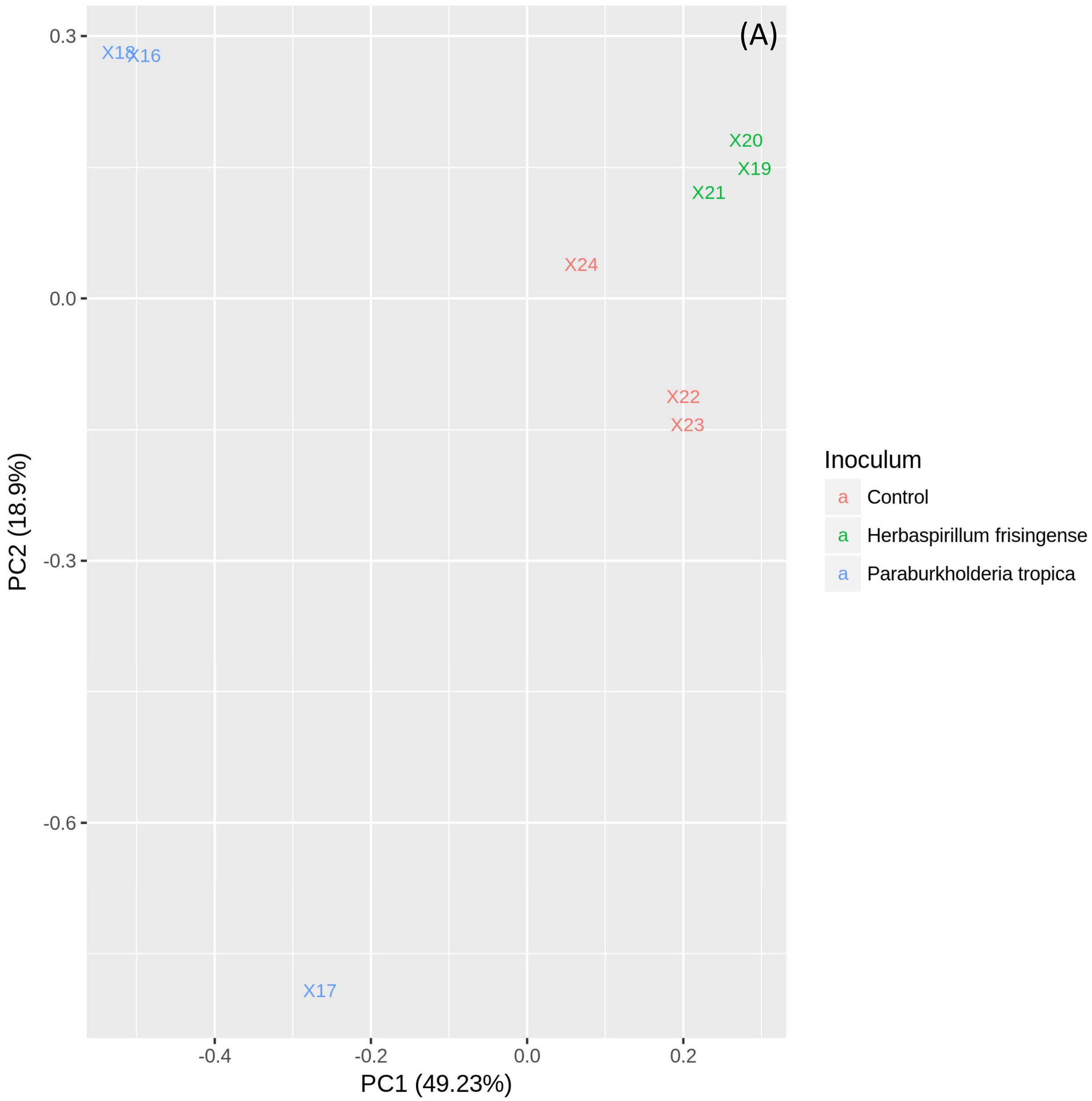
(A)
X18
X16
X20
X19
X21
X24
X22
X23
X17
0.3
0.0
-0.3
-0.6
-0.4
-0.2
0.0
0.2
PC2 (18.9%)
PC1 (49.23%)
Inoculum
Control
Herbaspirillum frisingense
Paraburkholderia tropica

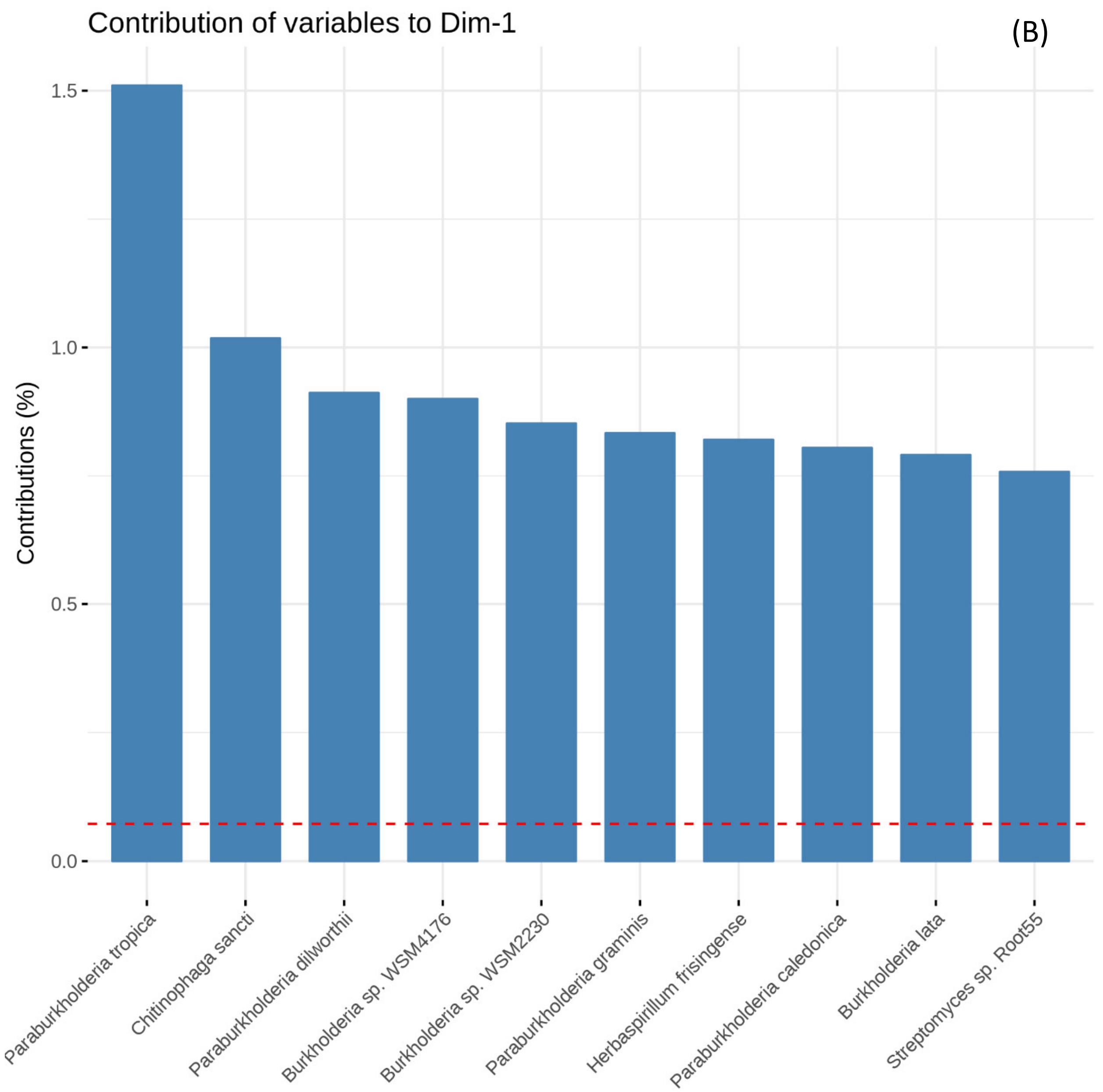
Contribution of variables to Dim-1
(B)
Contributions (%)
1.5
1.0
0.5
0.0
Paraburkholderia tropica
Chitinophaga sancti
Paraburkholderia dilworthii
Burkholderia sp. WSM4176
Burkholderia sp. WSM2230
Paraburkholderia graminis
Herbaspirillum frisingense
Paraburkholderia caledonica
Burkholderia lata
Streptomyces sp. Root55

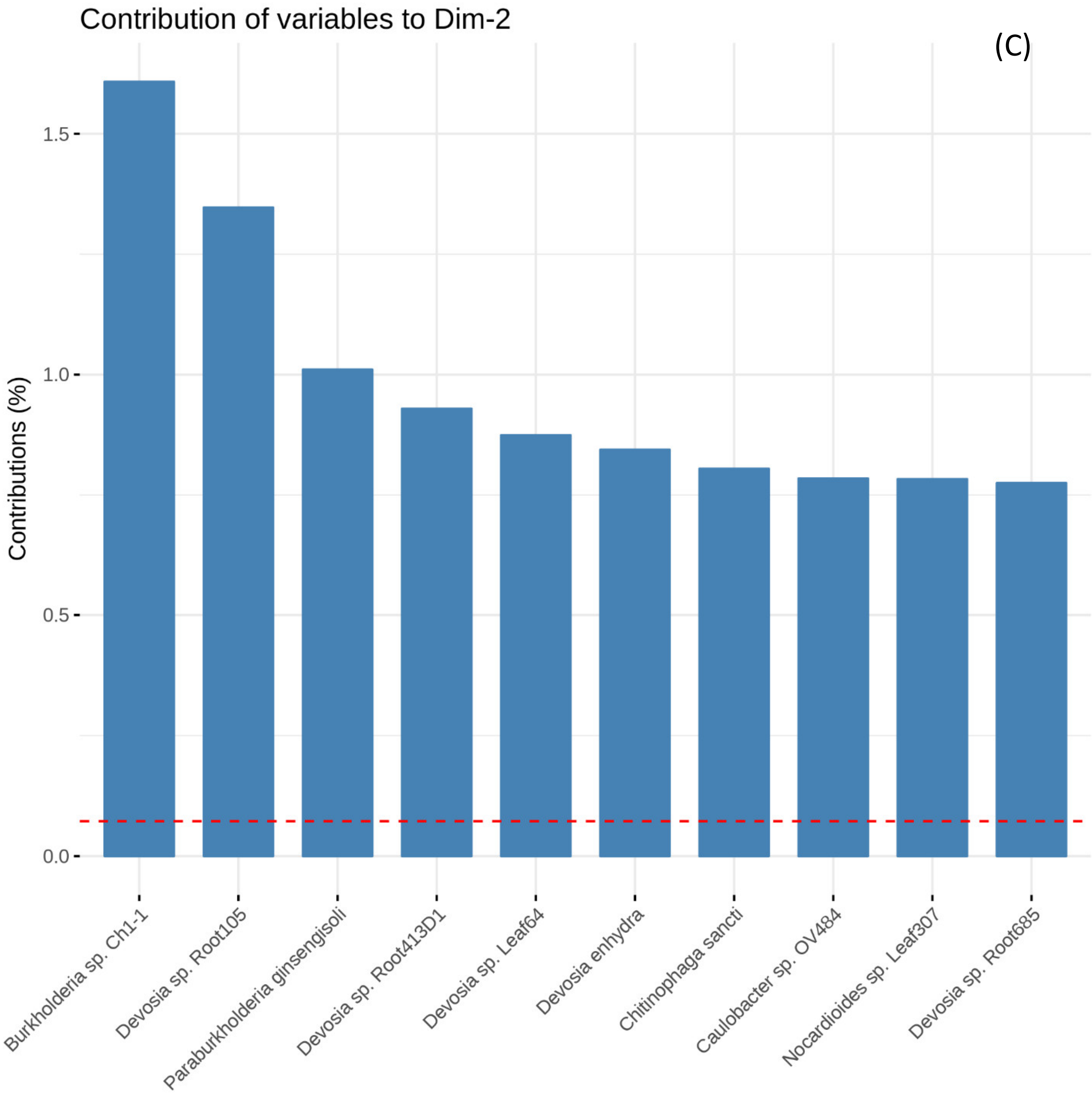

Figure S2. PCA analysis of the taxonomic classification at species level. A) PCA plot of the species abundance over all samples. B) Species contribution to PCA1. C) Species contribution to PCA2. Analysis was performed using the standard prcomp() function in R.

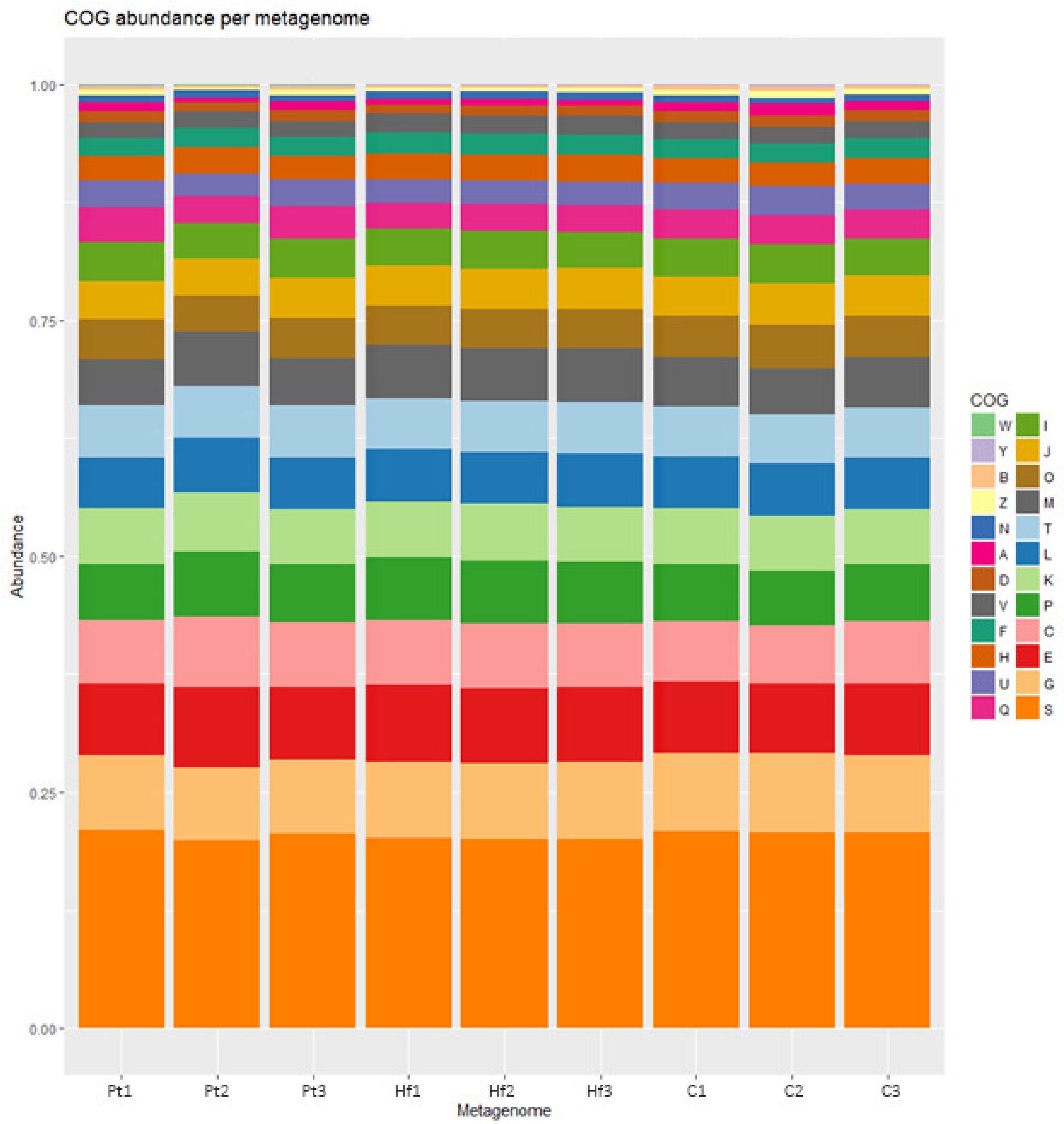

Figure S3. Abundance of COG terms per sample inoculated. Pt: *P. tropica* IAC/BECa 135 strain; Hf: *H. frisingense* IAC/BECa 152 strain; C: control; 1, 2, 3 are replicates. The value in each bar depicts the abundance of the COG in the metagenome. Definition of the COG categories are A: RNA processing and modification; B: chromatin structure and dynamics; C: energy production and conversion; D: cell cycle control and mitosis; E: amino Acid metabolis and transport; F: nucleotide metabolism and transport; G: carbohydrate metabolism and transport; H: coenzyme metabolism; I: lipid metabolism; J: translation; K: transcription; L" replication and repair; M: cell wall/membrane/envelop biogenesis; N: cell motility; O: post-translational modification, protein turnover, chaperone functions; P: inorganic ion transport and metabolism; Q: secondary structure; T: signal transduction; U: intracellular trafficking and secretion; V: defense mechanisms; Y: nuclear structure; Z: cytoskeleton; R: general functional prediction only; S: function unknown.

Table S4. Number of PGPR gene homologues identified in the genomes of *P. tropica* IAC/BECa 135 strain and *H. frisingense* IAC/BECa 152 strain using a manually compiled PGPR database (e-value >1e-102).

Category	Subcategory	Genes	Numeber of genes	
			H. frisingense	*B.tropica*
Hormones	ACC deaminase	*acdS*	80	183
Hormones	IAA	*aldH*	1	2
Nutrients	Nitrogen fixation	*nifB*	1	0
Nutrients	Phosphate Solubilization	*phoS*	1	0
Nutrients	Phosphate Solubilization	*pstB*	1	0
Hormones	IAA	*puuC*	1	0
Adaptation	T6SS	T6SS effectors	2	5
Adaptation	T6SS	*tssC*	1	2
Siderophore	Transport	*desA*	0	1
Siderophore	Transport	*fhuA*	0	1
Siderophore	Transport	*fhuB*	0	1
Nutrients	Nitrogen fixation	*nifS*	0	1
Nutrients	Nitrification	*pmoB*	0	3
Siderophore	Transport	*pvdE*	0	1

Table S5. Number of specific identified gene clusters.

	Anti-fungal	Anti-bacterial	Polysaccharide
P. tropica IAC/BECa 135	1	4	7
H. frisingense IAC/BECa 152	1	1	2

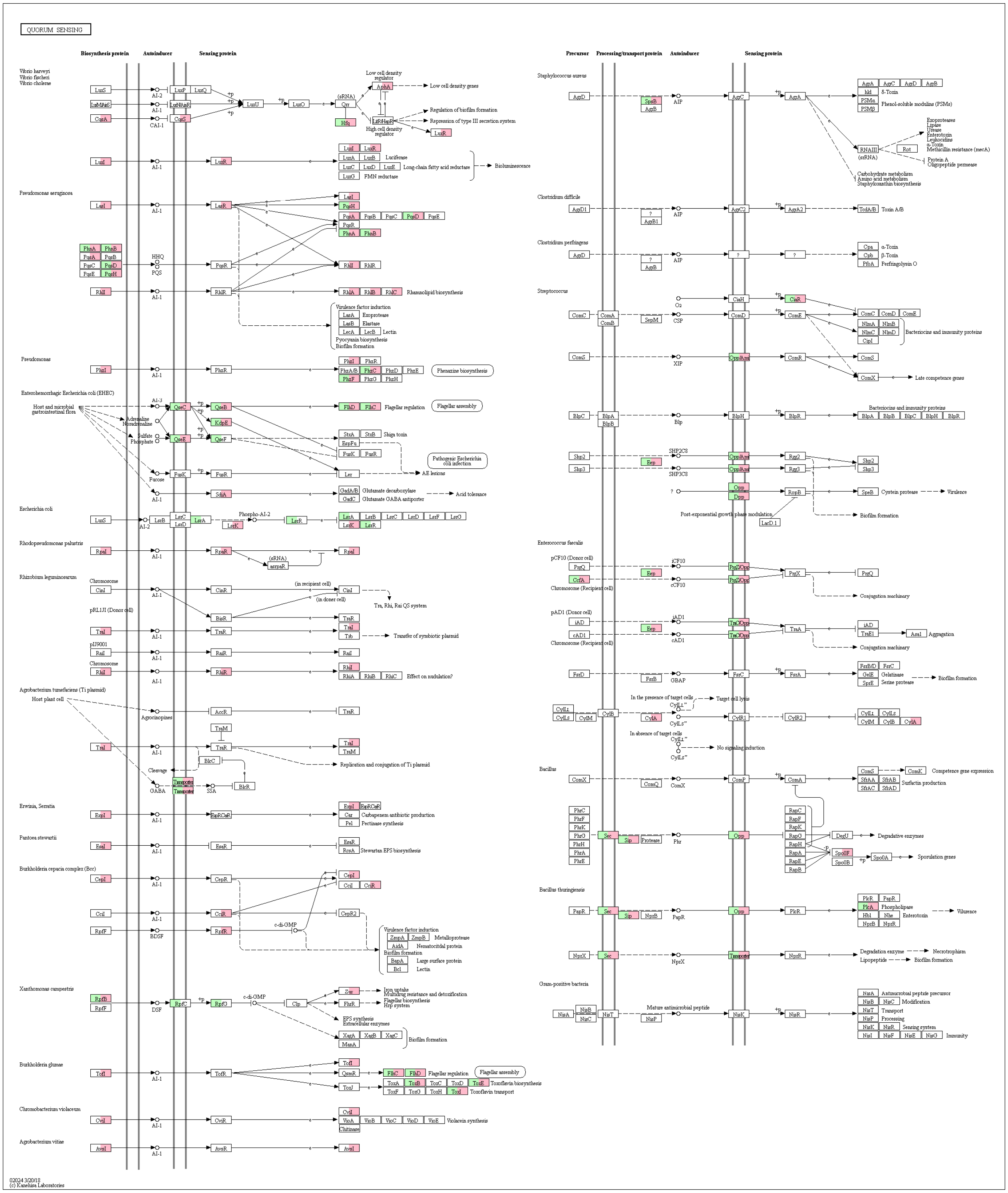

Figure S4. Quorum sensing KEGG. The enzymes in the pathways are colored based on presence in the genomes, where red means the enzyme is present in the *P. tropica* IAC/BECa 135 genome and green means the enzyme is present in the *H. frisingense* strain IAC/BECa 152 genome.

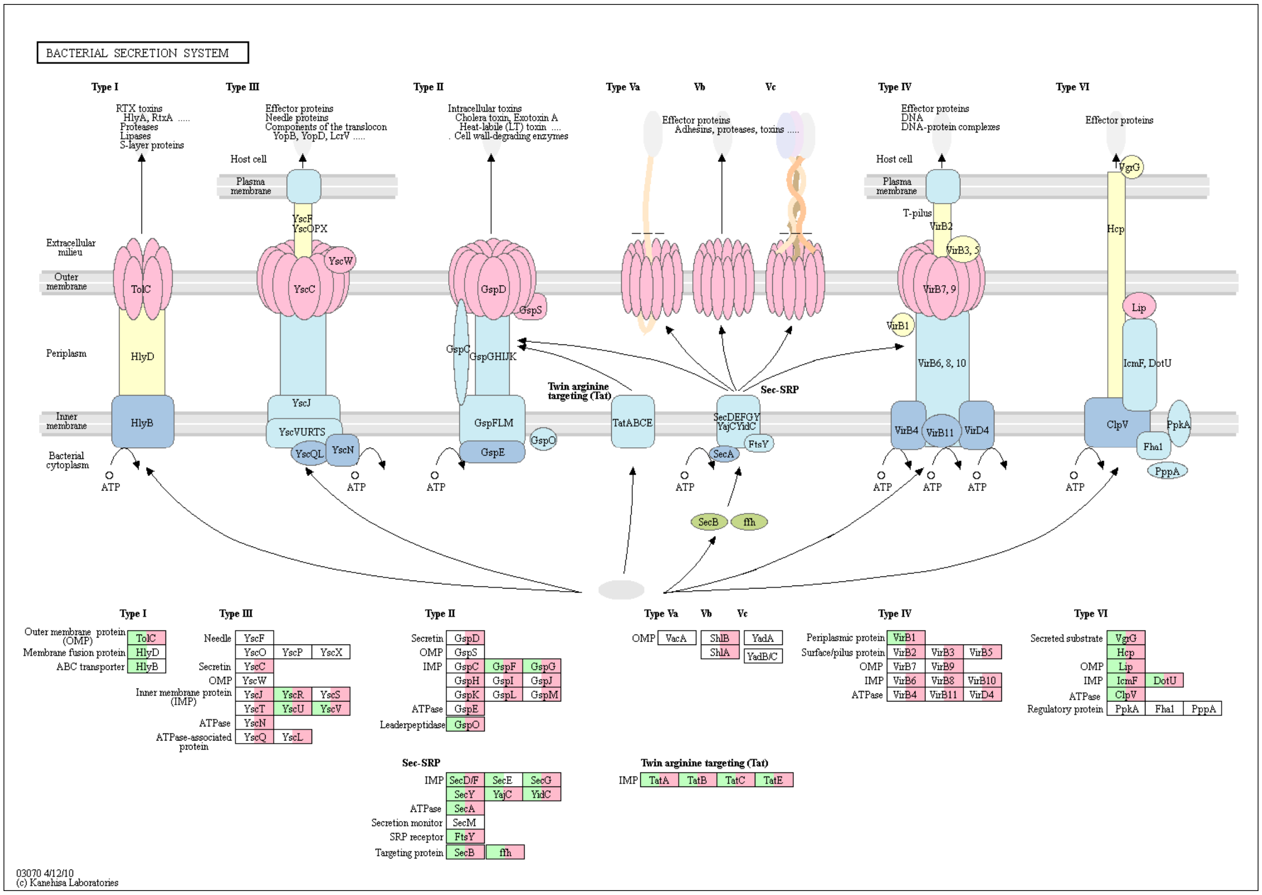

Figure S5. Bacterial secretion systems KEGG. The enzymes in the pathways are colored based on presence in the genomes, where red means the enzyme is present in the *P. tropica* IAC/BECa 135 genome and green means the enzyme is present in the *H. frisingense* strain IAC/BECa 152 genome.

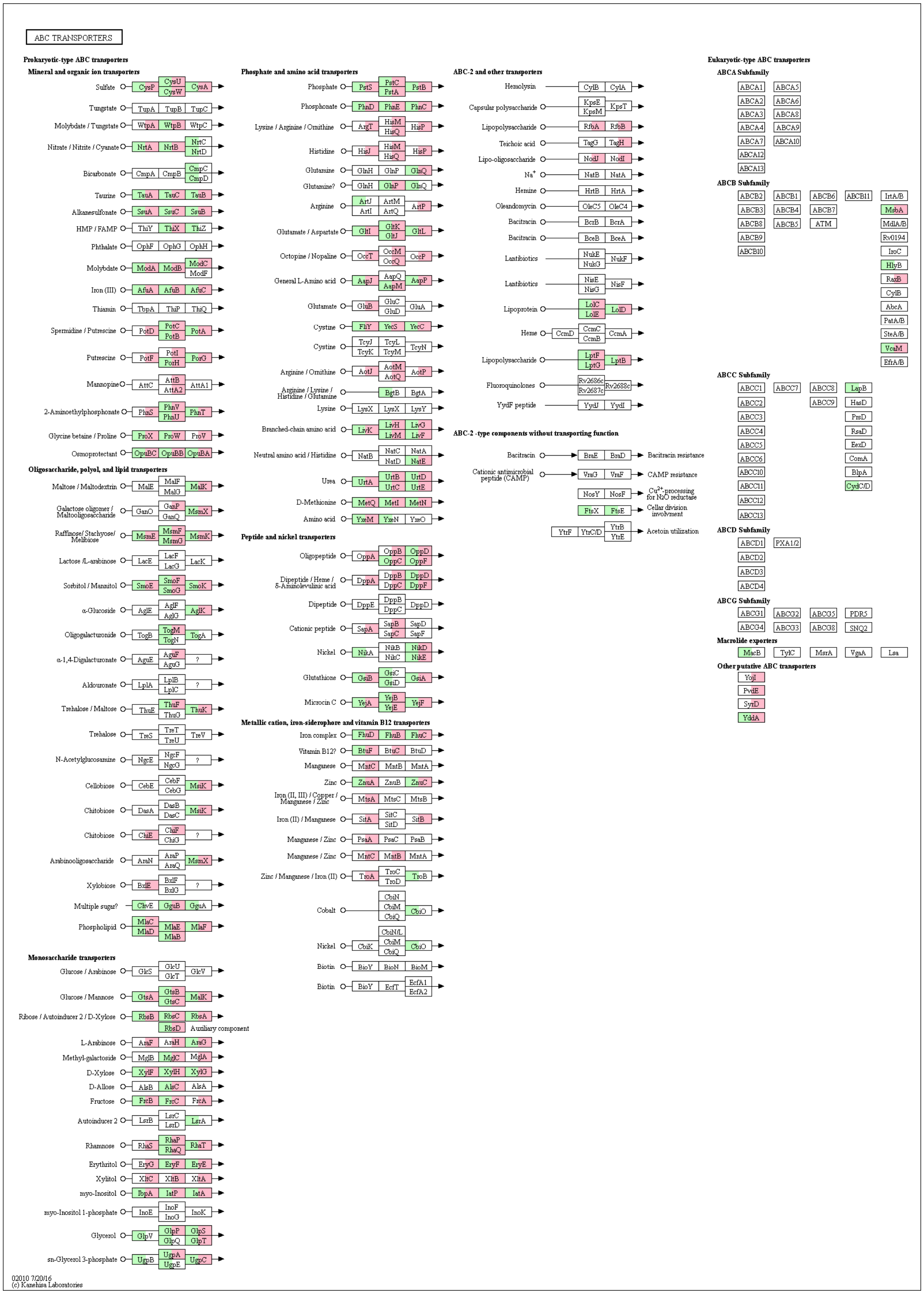

Figure S6. ABC transporters KEGG. The enzymes in the pathways are colored based on presence in the genomes, where red means the enzyme is present in the *P. tropica* IAC/BECa 135 genome and green means the enzyme is present in the *H. frisingense* strain IAC/BECa 152 genome.

1. Kanehisa, M.; Goto, S.; Sato, Y.; Kawashima, M.; Furumichi, M.; Tanabe, M. Data, information, knowledge and principle: back to metabolism in KEGG. *Nucleic acids research* **2013**, *42*, D199–D205.
